# Antimicrobial Evaluation of Mangiferin Analogues

**DOI:** 10.4103/0250-474X.56023

**Published:** 2009

**Authors:** S. K. Singh, Y. Kumar, S. Sadish Kumar, V. K. Sharma, K. Dua, A. Samad

**Affiliations:** D. J. College of Pharmacy, Niwari road, Modinagar-201 201, India; 1 ITS Paramedical College (Pharmacy), Muradnagar, Ghaziabad-201 206, India

**Keywords:** Antifungal, antimicrobial, *Mangifera indica*, mangiferin

## Abstract

The naturally occurring xanthone glycoside mangiferin has been isolated by column chromatography from the ethanol extract of stem bark of *Mangifera indica*. Mangiferin was further converted to 5-(N-phenylaminomethyleno)mangiferin, 5-(N-p-chlorophenylaminomethyleno) mangiferin, 5-(N-2-methylphenylaminomethyleno) mangiferin, 5-(N-p-methoxyphenylaminomethyleno) mangiferin, 5-(N, N-diphenylaminomethyleno) mangiferin, 5-(N--napthylaminomethyleno) mangiferin and 5-(N-4-methylphenylaminomethyleno) mangiferin. Mangiferin and its analogues were characterized by melting point and R_f_ value determination and through spectral technique like UV, IR, and NMR spectral analysis. The synthesized compounds were screened for antimicrobial activity.

Mangiferin, C_19_H_18_O_11,_ a glucoxanthone (1,3,6,7-tetrahydroxyxanthone-C_2_-β-D-glucoside) has been reported to be present in various parts of *Mangifera indica* viz leaves[1], fruits[2], stem bark[3], heartwood[4] and roots[5]. Mangiferin has attracted considerable interest in view of its numerous pharmacological activities, including antibacterial[6], antitumor, immunomodulatory and antiHIV[7], antidiabetic[8], antioxidative[9], anthelminthic and antiallergic[10], and antiinflammatory activity[11], antiviral[12], macrophage-inducing activity[13]. In Cuba, mangiferin is traditionally used as an antiinflammatory, analgesic and also as an antioxidant under brand name Vimang®. In Sri Lanka, mangiferin is used in the obesity treatment and particularly for diabetes type II under brand name Salaretin®. Updated literature survey reveals that many attempts have not been made to make the derivatives of mangiferin and consequently the derivatives of mangiferin have also not been subjected to the pharmacological screening. This prompted us to investigate upon mangiferin and its derivatives for their pharmacological screening.

The stem bark of *Mangifera indica* cultivar desi which was collected from saunda village Modinagar, Ghaziabad district of UP in the month of April 2006, was authenticated at the Department of Botany, M. M. P. G. College, Modinagar. Bacterial and fungal strains were obtained from the Institute of Microbial Technology, Chandigarh, India. Melting points were determined in open capillary tubes and purity of the compounds was checked by TLC on silica gel G. UV spectra were recorded on Systronics double beam UV spectrophotometer 2202, IR spectra were recorded in KBr on Jasco FTIR 4100 spectrophotometer, NMR spectra on Bruker avance II-400 MHz., spectrometer using TMS as internal reference. The bark was dried at room temperature and coarsely powdered. The fresh air-dried and coarsely powdered bark of *Mangifera indica* was extracted exhaustively with petroleum ether (60–80°) in Soxhlet apparatus to remove fatty matter for 56 h. Coarsely powdered bark of *Mangifera indica* was extracted exhaustively with ethanol (95%) in Soxhlet apparatus for 56 h. The combined alcohol extracts were concentrated under reduced pressure. Then, yellow amorphous powder was obtained.

The dried alcoholic extract was adsorbed on silica gel (60-120 mesh) and chromatographed over silica gel column packed in petroleum ether (60-80°). The column was eluted with chloroform:methanol (1:1) which gave mangiferin as a pale yellow amorphous powder. This upon crystallization from ethanol, produced pale yellow needle shaped mangiferin crystals, mp: 269-270°, R_f_: 0.77 using n-butanol:acetic acid:water (4:1:2.2) as a solvent system, λ max: 205.6, 256.8, 238.4, 315.2, 367.2 nm. IR (KBr) cm^−1^: 3366(O-H), 2937(C-H), 1649(>C=O), 1495(C=C), 1253(-C-O), 1050(C-O-C). NMR (δ ppm): 13.81(ArOH intramolecularly bonded, 1H), 7.9 (ArOH, 3H), 6.82 (Ar-H, 1H), 6.36 (Ar-H, 1H), 7.4 (ArH, 1H), 2.5 (-C-OH, 4H), 3.7 (-CH-O-, 2H), 3.3 (-CH-, 2H), 3.5 (-CH-, 3H).

The general method used for the preparation of mangiferin analogues is as follows; a mixture of equal mols of mangiferin, powdered paraformaldehyde and aromatic amine, 10 ml of 95% ethanol and 1 ml of concentrated hydrochloric acid was refluxed, cooled to room temperature and kept in a refrigerator overnight. The solid was filtered and washed with water and recrystallized from ethanol (Scheme 1).

**Scheme 1:**
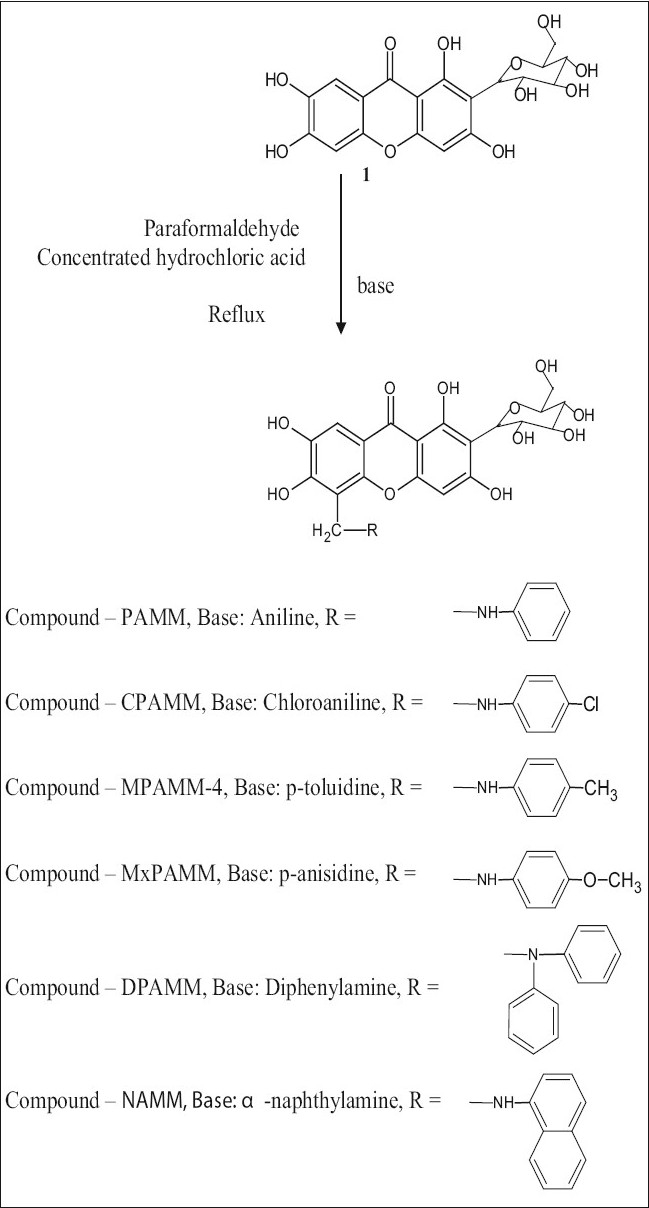
Synthesis of mangiferin analogues, 1 is mangiferin

5-(N-phenylaminomethyleno) mangiferin (PAMM); mp: 190°, R_f_: 0.60, λ max: 239.6, 261.2, 317.6, 370.4 nm. IR (KBr) cm^−1^: 3551(O-H), 3319(N-H), 2929(C-H), 1625(>C=O), 1488(C=C), 1383(-C-N), 1293(-C-O), 1037(C-O-C). NMR (δ ppm): 13.70 (ArOH intramolecularly bonded, 1H), 8 (ArOH, 3H), 6.82 (Ar-H, 6H), 7.39 (Ar-H, 1H), 3.7 (Ar-CH_2_ -N-, 2H), 4.1 (Ar-NH-, 1H), 2.9 (-C-OH, 4H), 3.7 (-CH-O-, 2H), 3.4 (-CH-, 1H), 3.5 (-CH-, 4H).

5-(N-p-chlorophenylaminomethyleno) mangiferin (CPAMM); mp: 210°, R_f_: 0.69, λ max: 225.2, 228.8, 261.2, 318.8, 368 nm. IR (KBr) cm^−1^: 3410(O-H), 3360(N-H), 2926(C-H), 1625(>C=O), 1429(C=C), 1375(-C-N), 1295(-C-O), 1079(C-O-C), 715(C-Cl). NMR (δ ppm): 13.66 (ArOH intramolecularly bonded, 1H), 7.9 (ArOH, 3H), 6.82 (Ar-H, 5H), 7.36 (Ar-H, 1H), 4.2 (Ar-CH_2_-N-, 2H), 4.0 (Ar-NH-, 1H), 2.1 (-C-OH, 4H), 3.7 (-CH-O-, 2H), 3.4 (-CH-, 5H).

5-(N-4-methylphenylaminomethyleno) mangiferin (MPAMM); mp: 195°, R_f_: 0.53, λ max: 230, 261.2, 317.6, 370.4 nm. IR (KBr) cm^−1^: 3493(O-H), 3483(N-H), 2971(C-H), 1638(>C=O), 1429(C=C), 1283(-C-N), 1044(C-O-C), 713. NMR (δ ppm): 13.66 (ArOH intramolecularly bonded, 1H), 7.9 (ArOH, 3H), 6.82 (Ar-H, 5H), 7.36 (Ar-H, 1H), 2.3 (Ar-CH_3,_ 3H), 3.7 (Ar-CH_2_-N-, 2H), 4.2 (Ar-NH-, 1H), 2.3 (-C-OH, 4H), 3.7 (-CH-O-, 2H), 3.3 (-CH-, 5H).

5-(N-p-methoxyphenylaminomethyleno) mangiferin (MxPAMM); mp: 190°, R_f_: 0.45, λ max: 210.8, 224, 261.2, 317.6, 370.4 nm. IR (KBr) cm^−1^: 3536(O-H), 3445(N-H), 2941(C-H), 1646(>C=O), 1432(C=C), 1283(-C-N), 1180(Ar-O-C), 1078(C-O-C). NMR (δ ppm): 13.66 (ArOH intramolecularly bonded, 1H), 7.9 (ArOH, 3H), 6.8 (Ar-H, 1H), 6.9 (Ar-H, 4H), 7.36 (Ar-H, 1H), 4.2 (Ar-CH_2_-N-, 2H), 4.0 (Ar-NH, 1H), 3.8(Ar-O-CH_3_, 3H), 2.1 (-C-OH, 4H), 3.8 (-CH-O-, 2H), 3.3 (-CH-, 5H).

5-(N, N-diphenylaminomethyleno) mangiferin (DAMM); mp: 210°, R_f_: 0.82, λ max: 257.6, 240.8, 305.6, 364.4 nm. IR (KBr) cm^−1^: 3371(O-H), 2931(C-H), 1647(>C=O), 1405(C=C), 1297(-C-N), 1253(-C-O), 1031(C-O-C). NMR (δ ppm): 13.78 (ArOH intramolecularly bonded, 1H), 7.87 (ArOH, 3H), 6.84 (Ar-H, 2H), 7.4 (Ar-H, 2H), 7.04 (Ar-H, 4H), 7.02 (Ar-H, 4H), 3.9 (Ar-CH_2_-N-, 2H), 2.1 (-C-OH, 4H), 3.7 (-CH-O-, 2H), 3.3 (-CH-, 2H), 3.4 (-CH-, 3H).

5-(N-α-napthylaminomethyleno) mangiferin (NAMM); mp: 205°, R_f_: 0.60, λ max: 244.4, 297.2, 306.8 nm. IR (KBr) cm^−1^: 3443(O-H), 3339(N-H), 2927(C-H), 1621(>C=O), 1482(C=C), 1385(-C-N), 1290(-C-O), 1038(C-O-C). NMR (δ ppm): 13.78 (ArOH intramolecularly bonded, 1H), 7.9 (ArOH, 3H), 6.87 (Ar-H, 1H), 7.36 (Ar-H, 1H), 7.4 (napth-H, 5H), 7.5 (napth-H, 2H), 4.29 (Ar-CH_2_-N-, 2H), 4.1(Ar-NH-, 1H), 2.1 (-C-OH, 4H), 3.8 (-CH_2_-O-, 2H), 3.4 (-CH-, 5H).

Antimicrobial evaluation was determined using the disc diffusion method[14]. Bacterial strains of *Staphylococcus aureus* subsp. aureus (MTCC-737) and *Escherichia coli* (MTCC-1687) and fungal strains of *Candida albicans* (MTCC-183) and *Aspergillus niger* (MTCC-228) were used. The nutrient agar plates were prepared by pouring 15 ml of molten media into sterile Petri plates. The plates were allowed to solidify for 5 min and 0.1% inoculum suspension was swabbed uniformly and the inoculum was allowed to dry for 5 min. The compounds were loaded on 5 mm discs. The loaded discs were placed on the surface of medium and the compounds were allowed to diffuse for 5 min and the plates were kept for incubation at 37° for 24 h for bacteria and 30° for 48 h for fungi with yeast peptone dextrose agar and czapek yeast agar media. At the end of incubation, inhibition zones formed around the discs were measured (Table 1).

**TABLE 1:** ANTIBACTERIAL AND ANTIFUNGAL ACTIVITY OF COMPOUNDS

Compound	Inhibition zone diameter (mm)			
	
	S. a.	E. c.	C. a.	A. *n*.
MG	10	8	12	11
PAMM	7	8	9	10
CPAMM	12	12	10	10
MPAMM-4	8	14	11	15
MxPAMM	8	8	10	10
DPAMM	12	8	10	9
NAMM	10	8	15	11
Ampicillin	18	17	--	--
Clotrimazole	--	--	20	17

*S. a.* is *Staphylococcus aureus*, *E. c.* is *Escherichia coli*, *C. a.* is *Candida albicans* and *A. n.* is *Aspergillus niger*, Control (DMF) = no activity. Both, test compound and standard were tested at 30 μg/ml.

In the process of isolation of mangiferin, stem bark of *Mangifera indica* was defatted with petroleum ether (60-80°) prior to extraction with ethanol 95%. The extract was chromatographed over silica gel and eluted with chloform:methanol (1:1) to afford the parent mangiferin as pale yellow needle shaped crystals. Mangiferin analogues such as PAMM, CPAMM, MPAMM, MxPAMM, DAMM and NAMM were synthesized. The synthesized mangiferin analogues were characterized by R_f_, mp, UV, IR and NMR spectral analyses. The absorbed maxima 205.6, 256.8, 238.4, 315.2 and 367.2 nm of mangiferin is closely related to that of reported UV spectral data[15]. Mangiferin and its derivative were also confirmed by proton NMR signals. Mangiferin and its analogues were subjected to antimicrobial study. From the extent of zone of inhibition, the activities were compared. All the analogues exhibited moderate to mild activity against *Staphylococcus aureus, Escherichia coli, Candida albicans, Aspergillus niger.* One of the mangiferin analogues namely MPAMM was found to be more effective than other compounds against Gram-negative organism, *Escherichia coli*. In the antifungal activity study, mangiferin and NAMM were found to be more effective than other compounds against *Candida albicans.* Good activity against *Aspergillus niger* was shown by 5-(N-4-methylphenylaminomethyleno) mangiferin.
